# Nutrition: the missing link in the battle against microbial resistance?

**DOI:** 10.7189/jogh.09.010321

**Published:** 2019-06

**Authors:** Stefan A Unger, Henry Mark, Claudia Pagliari

**Affiliations:** 1 University of Edinburgh, Department of Child Life and Health, Edinburgh, UK; 2 Royal Hospital for Sick Children, Department of Respiratory Medicine, Edinburgh, UK; 3 International Development Consultant in Nutrition, Nottingham, UK; 4 University of Edinburgh, Usher Institute of Population Health Sciences and Informatics, Edinburgh, UK; * Equal authorship

The growing threat posed by antimicrobial resistance (AMR) continues to attract scientific and policy attention [1,2]. A number of recent publications have reignited the debate on striking a balance between the use of antibiotics as a powerful public health tool [3] and the significant risks posed by AMR, including an increase in mortality from drug-resistant strains of common bacterial infections in children with HIV, TB and malaria [4].

Rochford and others recently called for an international inter-agency effort to strengthen global governance of AMR [1]. They suggest setting up a multi-stakeholder Global Steering Board, within existing organisational structures, to develop a legal agreement on international standards and norms with the following four key objectives: 1) ensuring the appropriate use of antibiotics in humans and animals, 2) eradicating untreated effluent, 3) strengthening infection prevention and control measures such as water, sanitation, and hygiene programmes; and 4) providing appropriate access to a regulated supply of quality-assured affordable antimicrobials. We agree with all of these points, but would argue that an important contribution to AMR prevention should be added to these objectives: the promotion of good nutrition. Here we draw on indirect evidence and theory to support this proposition.

**Figure:**
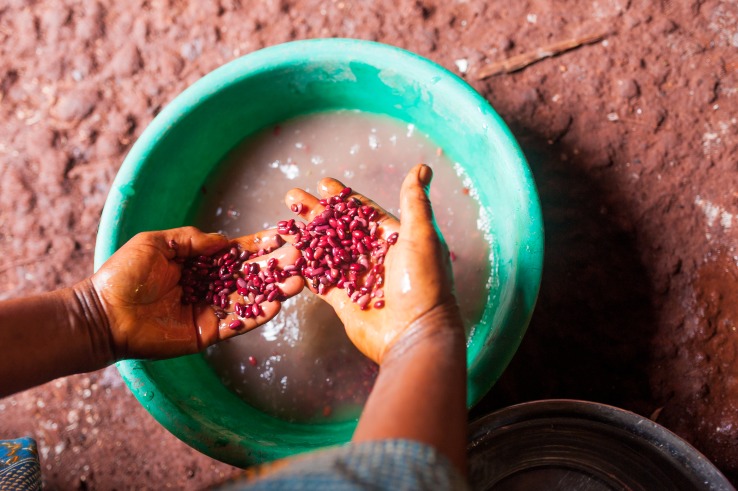
Photo: iStock.

Following previous observations in Ethiopia of a possible reduction in childhood mortality resulting from antibiotic interventions tackling trachoma, the New England Journal of Medicine published details of a multi-country cluster-randomised trial in Sub-Saharan Africa, providing twice-yearly mass administration of azithromycin vs placebo to children aged 1 to 59 months [3]. A total of 190 238 children from 1533 randomized communities in Malawi, Niger and Tanzania were identified at baseline and 323 302 person-years were monitored. Communities were included if they were not eligible for mass distributions of azithromycin for trachoma and none of the children had previously received azithromycin. Limited additional information was collected on each individual child and community. Coverage with azithromycin (approximately 20 mg per kilogram of body weight) and placebo over four twice-yearly distributions was around 90%. The primary outcome was an aggregate of all-cause mortality with country-specific rates as subgroup analyses. The results of this study showed an overall 13.5% reduction in all-cause mortality in the azithromycin group but with wide confidence intervals (95% confidence interval (CI) = 6.7 to 19.8).

An editorial in the *Lancet Infectious Diseases* highlighted that the mortality reduction was highest in Niger at 18.1% (95% CI = 10.0 to 25-5; *P* < 0.001) while there were non-significant reductions of 5.7% (95% CI = -9.7 to 18.9, *P* = 0.45) in Malawi and 3.4% (95% CI = -21.2 to 23.0, *P* = 0.77) in Tanzania [5]. While the lack of data on the participants makes it hard to interpret these differences, we believe a key contextual influence which may be responsible is the nutritional status of children under 5 in the three cohorts. Although all three countries have poverty levels of 81% and above[6], the nutritional status of these populations differs widely. According to the latest estimates from UNICEF the prevalence of wasting is 10.3% in Niger–where a large reduction in mortality was observed–while in Malawi and Tanzania it is much lower at 2.8% and 4.5%, respectively [7].

Epidemiological data has shown that malnutrition increases the risk of infectious morbidity and mortality in childhood.[8] Our examination of over 21 000 acute presentations to a rural primary health care station in West Africa, demonstrated that wasting, indicated by a weight-for-height Z-score (WHZ)<-2, accounted for 21% of severe illness and that when the analysis was extended to those *at risk* of wasting (<-1 WHZ) this further increased to 35% [9]. Our findings suggest that children who are not acutely malnourished by definition, and are therefore ineligible for community-based feeding programmes and other therapeutic interventions, are still significantly more vulnerable to severe infectious disease than better nourished children.

**Figure 1:**
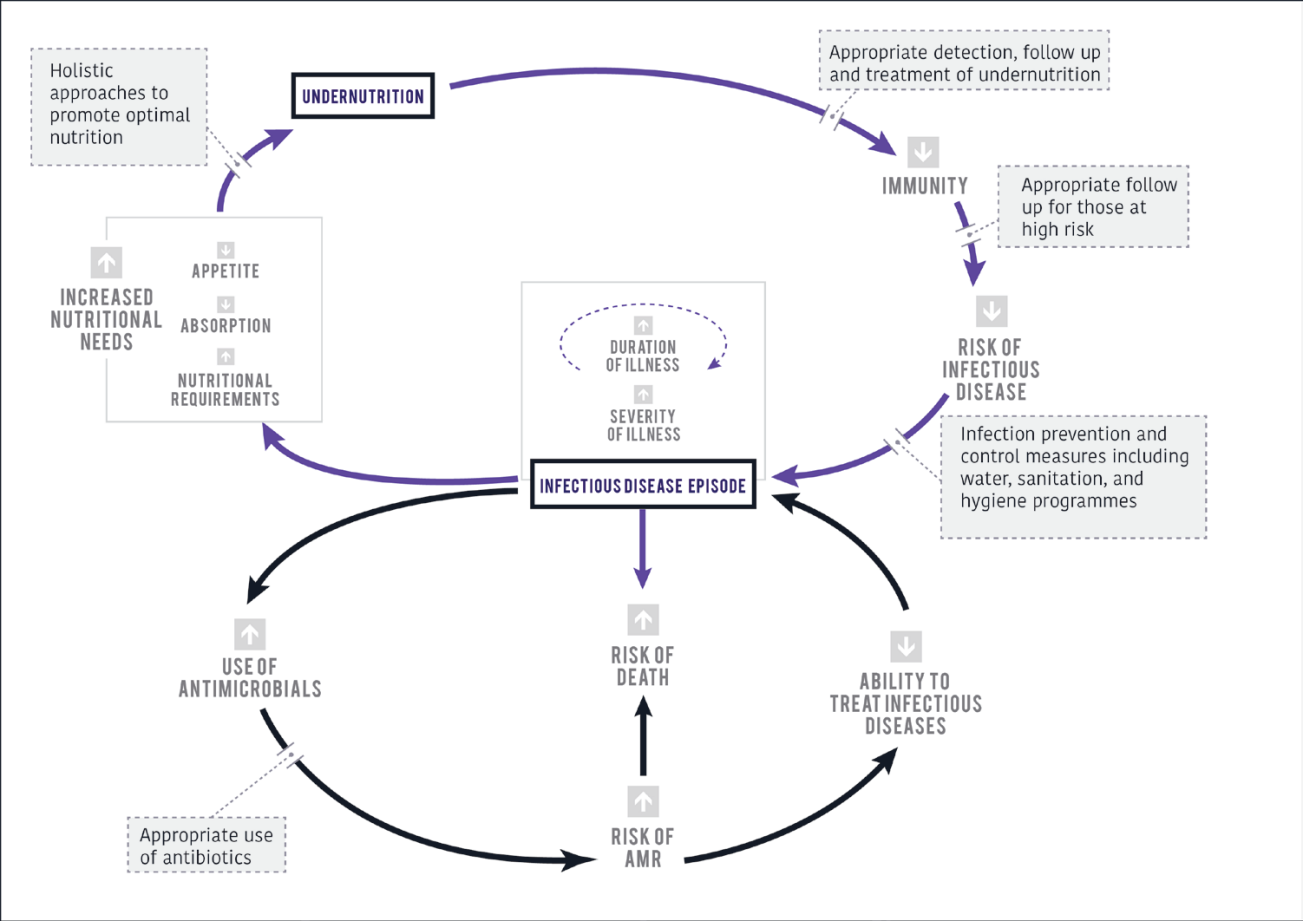
The relationship between undernutrition, infectious disease episodes and risk of antimicrobial resistance (AMR). Blue arrows represent the cycle of immunity, infectious disease risk and nutritional needs. Black arrows indicate the associated AMR risk. Grey boxes indicate key interventions required at different points in the cycle to reduce malnutrition, infectious disease risk and AMR risk. Malnutrition is both a cause and consequence of infectious disease episodes and can affect the severity and duration of infectious disease episodes. Holistic approaches to both tackle malnutrition and promote optimal nutrition can have an upstream influence on both infectious disease and AMR by reducing the need for antibiotic use.

We acknowledge that the relationship between infection and malnutrition is complex (**Figure 1**) [10]. Trials of blanket preventative nutritional supplementation have shown limited and heterogeneous effects on growth and development while concerns have also been raised about the potential long-term health risks of processed food supplementation [11]. Even when targeting children presenting with an acute illness, short-term supplementation with small quantities of micronutrient fortified lipid-based supplements and ready-to-use therapeutic foods appears to have only limited effects on subsequent growth, future severe malnutrition or illness episodes [11].

Despite these complexities, it is reasonable to argue that better nourished children will be less susceptible to infectious diseases and thus less likely to require antibiotics. A similar logic was given by Laxminarayan et al in their paper ‘*Antibiotic resistance – the need for global solutions’* [12], who speculate that the effects of AMR are likely to be higher in vulnerable groups, including those who are malnourished. *We therefore propose that strategies for global antibiotic stewardship should include interventions to both tackle malnutrition and promote optimal nutrition as an upstream influence on both infectious disease and AMR.*

Given the need for AMR strategies to integrate multiple disease prevention tactics, including interventions to improve hygiene and child feeding practices, holistic, person-centred, multi-layered approaches are most likely to deliver lasting results. We suggest that all health care encounters, at community, primary or secondary levels provide a unique opportunity to: 1) offer nutritional education, to tackle low awareness or cultural barriers [13]; 2) provide targeted nutritional interventions, including treatment for malnourished children [13], 3) provide guidance on hygiene promotion [14] and symptom recognition for severe infectious diseases [15]; and 4) diagnose and treat infections appropriately [16]. Such a combined approach may provide mothers and carers with the tools required to reduce the incidence of infectious disease, decrease time to recovery and lower the risk of re-infection, thus reducing the need for antibiotics, and, by extension, helping to combat AMR.

Mobile phone-based (mHealth) tools have potential to support the delivery of such holistic interventions as well as to collect data on nutritional and infection indicators to guide the targeting of interventions and enable smarter AMR management [17]. While holding much promise for improving population health, such multifaceted approaches require strong country-level commitment and community engagement [18]. At the same time, global political solutions and agreements are needed to ensure the fair and secure distribution of food, rather than focusing on crisis-driven supplementation.

We strongly recommend that nutrition be considered as an essential element of strategies to provide antibiotic stewardship and security. Active and preventative, patient-centred nutritional interventions within primary health care and communities have a key role to play in addressing the global challenge of AMR, supported by further research to unpick the complex relationship between nutrition, infection and AMR.
